# Second-line treatment of recurrent HNSCC: tumor debulking in combination with high-dose-rate brachytherapy and a simultaneous cetuximab-paclitaxel protocol

**DOI:** 10.1186/s13014-016-0583-0

**Published:** 2016-01-20

**Authors:** M. Ritter, I. U. Teudt, J. E. Meyer, U. Schröder, G. Kovács, B. Wollenberg

**Affiliations:** Department of Otorhinolaryngology, Head and Neck Surgery, University Hospital Schleswig-Holstein Campus Lübeck, Ratzeburger Allee 160, 23538 Lübeck, Germany; Department of Otorhinolaryngology, Head and Neck Surgery, Asklepios Hospital Altona, Paul-Ehrlich-Straße 1, 22763 Hamburg, Germany; Department of Otorhinolaryngology, Head and Neck Surgery, Asklepios Hospital St.Georg, Lohmühlenstraße 5, 20099 Hamburg, Germany; Interdisziplinary Brachytherapy Unit, University Hospital Schleswig-Holstein Campus Lübeck, Ratzeburger Allee 160, 23538 Lübeck, Germany

**Keywords:** Brachytherapy, Cetuximab, HNSCC, Paclitaxel, Recurrent disease, Second-line therapy

## Abstract

**Background and purpose:**

After the failure of first-line treatment, the clinical prognosis in head and neck cancer (HNSCC) deteriorates. Effective therapeutic strategies are limited due to the toxicity of previous treatments and the diminished tolerance of surrounding normal tissue. This study demonstrates a promising second-line regimen, with function preserving surgical tumor debulking, followed by a combination of postoperative interstitial brachytherapy and a simultaneous protocol of cetuximab and taxol.

**Patients and methods:**

From January 2006 to May 2013, 197 patients with HNSCC were treated with brachytherapy at the University Hospital Schleswig-Holstein Campus Lübeck, including 94 patients due to recurrent cancer. Within these, 18 patients were referred to our clinic because of early progressive disease following first- or second-line treatment failure. They received the new palliative regimen. A matched-pair analysis including recurrent tumor stage, status of resection margins, tissue invasion and previous therapy was performed to evaluate this treatment retrospectively. Overall survival (OS), disease-free survival (DFS), functional outcome and treatment toxicity was analyzed on the basis of medical records and follow-up data.

**Results:**

DFS and OS of the study group were 8.7 and 14.8 months. Whereas, DFS and OS of the control group, treated only by function preserving tumor debulking and brachytherapy, was 3.9 and 6.1 months respectively. This demonstrates a positive trend through the additional use of the cetuximab-taxane protocol. Furthermore, no increase of therapy induced toxicities was displayed.

**Conclusion:**

Pre-treated patients with a further relapse benefit from the ‘cetuximab-taxane recurrency scheme’. It seems to be a valuable complement to interdisciplinary and multimodal tumor therapy, which improves OS and results in acceptable toxicity.

**Electronic supplementary material:**

The online version of this article (doi:10.1186/s13014-016-0583-0) contains supplementary material, which is available to authorized users.

## Full text article

### Background and purpose

Treatment options for locally recurrent or persistent head and neck squamous cell carcinoma (HNSCC) are limited. Depending on initial therapy and extent of the disease, treatment with curative intent, e.g. salvage surgery or further radiation, is occasionally feasible. However, in most cases recurrent disease is incurable and treated with a palliative approach [1].

Reported studies of re-irradiation with concurrent chemotherapy show high rates of locoregional control (LC) (25-30 %) and improved overall survival (OS) (10-30 % at 2 years), dependent on patient selection and treatment regimen. But in comparison with studies using chemotherapy alone, they demonstrate substantial toxicity (grade III-IV toxicities up to 40 % and mortality up to 10 %) [2–5].

Single-agent platinum chemotherapy stays the common standard for locally advanced recurrences. The response rate (RR), meaning the percentage of patients whose cancer shows a partial or complete tumor response, is estimated at 15 to 30 %. Disease-free survival (DFS) averages at 3 to 5 months and mean OS at 5 to 7 months [1, 6]. Aggressive multi-agent platinum combinations demonstrate enhanced RR up to 40 %. However, mean OS remains low with an average of 9 months [7, 8].

If the tumor progresses under first-line treatment or recurs again after initial complete response (first-line failure), the prognosis is even worse. RR to further chemotherapy decreases to 3 % and OS is about 3.5 months [1]. The sole treatment options are palliative chemotherapy, targeted therapy in the framework of clinical trials and best supportive care [9]. Recommended treatment strategies are single agent chemotherapy with taxane, platinum derivate, methotrexate or fluorouracil [10].

These patients are often debilitated and less able to tolerate further aggressive treatment. So the indication for additional second-line therapy has to be very stringent. Particular attention has to be paid to the side effect profile. Here a promising treatment regimen is reported and analyzed, containing function preserving surgical debulking, postoperative interstitial brachytherapy (BT) and a simultaneous protocol of cetuximab and paclitaxel.

### Patients and methods

From January 2006 to May 2013, we retrospectively reviewed 94 patients with recurrent head and neck cancer treated with postoperative interstitial high-dose-rate brachytherapy (HDR-BT) at the University Hospital Schleswig-Holstein Campus Lübeck. The study group, who was treated with our new multimodal therapy scheme, had developed progressive disease under or within a short time after first- or second-line therapy. Palliative treatment was indicated in patients with advanced locoregional disease, failing response to (radio)chemotherapy or exhausted radiation dose.

We included consecutive patients with histologically confirmed recurrent cancer without evidence of distant metastases and whose tumor was feasible to treat with BT. All patients had adequate renal, liver and hematological functions and were in eligible general condition, without concomitant malignancies or serious illness. Thus patients included were suitable for general anesthesia. Patients with known incompatibilities, of more than grade III towards cetuximab or taxol, were excluded. The decision towards the new scheme as an individual treatment was made in our interdisciplinary tumor board and discussed intensively with the patient. Informed consent was taken for this individual treatment plan.

### Chemotherapy

**Fig. 1:**
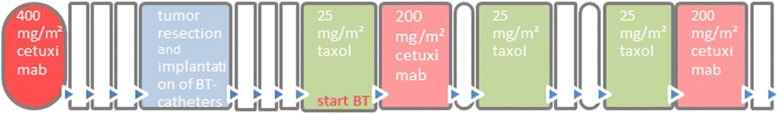
The cetuximab-taxane recurrency scheme. Shows a sketch of the cetuximab-taxan recurrency scheme and its time line. The regimen consists of operative debulking, postoperative brachytherapy and the simultaneous use of cetuximab and taxol. Brachytherapy was applied with a single dose of 2.5 Gy twice daily to a total dose of average 27.0 Gy

The ‘Cetuximab-Taxane Recurrency Scheme’ is based on the findings of Bonners Extreme trial. Bonner et al. reached a significant increase in LC and OS in locally advanced HNSCC by adding cetuximab to radiation [11]. Especially in platinum-refractory or -resistant disease, it seems to be a favorable option [12]. Vermorken et al. confirmed these findings in recurrent HNSCC by adding cetuximab to chemoradiation [13]. The guidelines of the National Comprehensive Cancer Network (NCCN) included cetuximab into standard regimes of locally advanced or recurrent head and neck cancer in 2011 (http://oralcancerfoundation.org/treatment/pdf/head-and-neck.pdf). We applied cetuximab according to Shin et al. to reach sufficient saturation in the tumor tissue [14]. Starting with a loading dose of 400 mg/m^2^, one week before tumor resection and implantation of the radiation catheters, cetuximab was applied with a dose of 200 mg/m^2^ once a week thereafter (Fig. 1).

Paclitaxel was administered only during fractionated radiation. Taxanes have been studied in combination with chemotherapy regimens. By adding docetaxel to cisplatin fluorouracil (PF) induction chemotherapy RR and OS were improved by showing a favorable toxicity profile [15, 16]. Particularly higher RRs between 20 and 43 % were reported [6]. In our regimen, taxol was given every three days during BT to achieve higher radiation sensitivity. The dosage of 25 mg/m^2^ was less than in other tumor protocols e.g. breast cancer.

To prevent anaphylaxis, chemoradiation was applied in combination with prednisolon 250 mg (2 h before), clemastin 2.68 mg and ranitidin 50 mg (1 h before). In addition, antibiotics and calcium were administered prophylactically to reduce perioperative infection and radiation damage.

### Surgery and BT-catheter placement

The tumor resection, reconstruction and one-step BT-catheter placement were all applied interdisciplinary by an experienced head and neck surgeon, under the surveillance of a BT expert.

### Brachytherapy

**Fig. 2:**
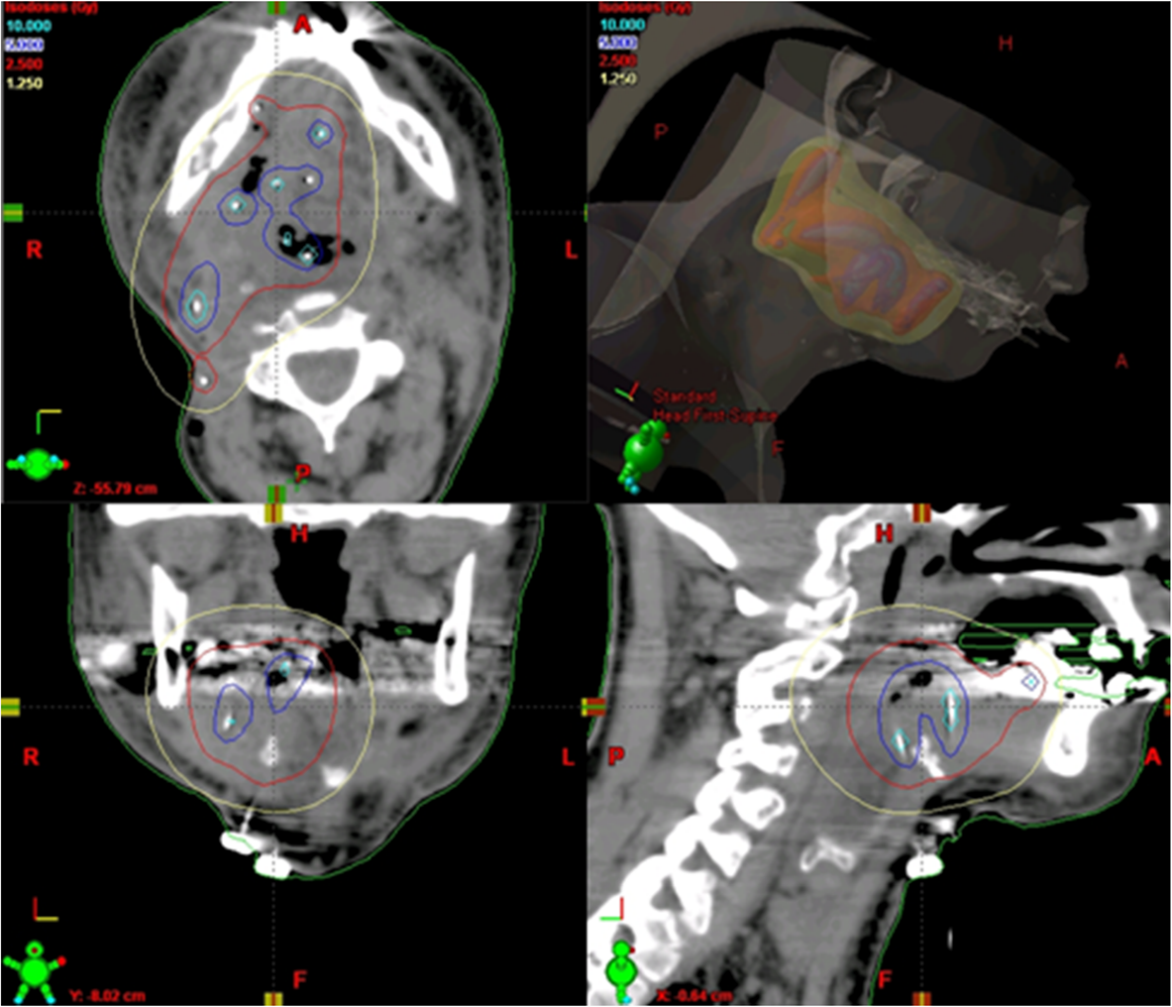
Dose distribution. Illustrates a thin slice computer tomography based 3D model of the implant, with optimized individual dose distribution to the target volume (V_100_ = within the red isodose). Hot (V_150_ = within the blue isodose) and cold (V_50_ = within the yellow isodose) spots were set according to biological needs: to areas of residual macroscopic tumor we applied higher local doses and organs at risk received less than the reference dose

For BT treatment planning purposes, thin slice computer tomography imaging was performed to control catheter placement and to create a 3D model of the implant. An individual optimized dose distribution to the target volume was calculated respecting a possible previous or adjuvant tissue radiation dose. Hot and cold spots were set according to biological needs: to areas of residual macroscopic tumor we applied higher local doses (up to 200 % of the reference isodose) and organs at risk were receiving less than the reference dose (Fig. 2).

BT was processed hypofractionated accelerated (unit dose 2.5 Gy) twice a day, with a minimum time interval of at least 6 hours between both fractions. Iridium-192 was used as the radiation source (370 GBq nominal activity) delivered with the Flexitron® (Elekta) afterloading machine.

### Early and late toxicities

Documentation of early toxicities was based on the modified CTC (common toxicity criteria) -classification of the German Cancer Society [17]. Late toxicities were classified by LENT (Late Effects Normal Tissue)-SOMA-classification of the National Cancer Institute [18]. All patients were followed up regularly, in both departments of otorhinolaryngology and brachytherapy.

### Statistics and analysis

For analysis three groups were compiled: the total group (*n* = 94), the study group (*n* = 18) receiving the cetuximab-taxane recurrency scheme and the matched pair control group (*n* = 18).

In advance, appropriate variables had to be found to create a control group with comparable prognosis. Within the total group DFS was associated with previous radiation (*p* = 0.004), tumor size (*p* = 0.025), UICC stage (*p* = 0.025), tissue invasion as lymphangiosis carcinomatosa and extracapsular spread (*p* = 0.001). OS was correlated with DFS (*p* = 0.001), T stage (*p* = 0.038) and lymphangiosis carcinomatosa (*p* = 0.012).

Brockstein proposes T stage, N stage and histological differentiation to be the critical factors to affect DFS or OS, whereas Vermorken considers tumor cell differentiation, ECOG performance score, weight loss, location of the primary tumor and prior radiotherapy to be essential [6, 19].

Based on this information, we created the control group by matched-pair analysis according to recurrent tumor stage, location of primary tumor, resection margin status, extend of tissue invasion and previous radiation. We proved congruence and relevant differences by chi-square test and Mann–Whitney U test.

To evaluate our regimen, study group, matched pair control group and total group were compared by OS, DFS, functional outcome and treatment toxicity. A Kaplan-Meier estimator was used to analyze the data statistically. Considering the matching process we also used McNemar’s test and a pared sample t-test to compare OS and DFS of the study group and the control group. In a subgroup analysis we proved the impact of further prognostic factors to our data. Statistics were calculated using IBM SPSS Statistics 21, supported by our department of statistics and biometrics.

## Results

### Patient and tumor characteristics

All patient and tumor characteristics are summarized in Table 1. A total of 18 patients, 12 men and 6 women, at a median age of 48.0 (range 45–68) were enrolled in the treatment arm. Twelve (66.7 %) patients suffered from advanced recurrent disease (UICC stage III-IV), due to a higher T-stage (55.6 %) or advanced nodal disease (N1-N2 16.7 %, N3 11.1 %). Five (27.8 %) patients with local disease (UICC stage I-II) were treated for lymphangiosis carcinomatosa, vessel infiltration, perineural infiltration or positive resection margins.

**Table 1:** Patient and tumor characteristics

		Total group *(n = 94)*			Study group *(n = 18)*			Control group *(n = 18)*			*p*-values*
		*n*	%	value (range)	n	%	value (range)	n	%	value (range)	
	Age < 60 years	36	38.3		13	72.2		5	27.8		
	Age ≥ 60 years	58	61.7		5	27.8		13	72.2		
	Age at onset of recurrence disease (years)			62.0 (53–71)			48.0 (45–68)			66.5 (57–73)	(0.006)
	Time to first recurrence < 3 months	10	10.3		9	50.0		0	0		
	Time to first recurrence ≥ 3 months	81	83.5		6	33.3		18	100		
	Previous recurrence free survival (months)			24.0 (10–73)			2.3 (2–8)			38.1 (18–99)	(0.003)
	First recurrence	49	52.1		9	50.0		7	38.9		
	Several recurrences	45	47.9		9	50.0		11	61.1		
Previous treatment	Operation	72	76.6		12	66.7		13	72.2		(0.177)
	Chemotherapy	25	26.1		6	33.1		8	44.0		(0.978)
	Radiotherapy	63	67.0		15	83.3		17	94.4		(0.584)
	Previous total radiation dose (Gy)			64.2 (33–105)			68.1 (50–105)			66.2 (59–77)	(0.696)
rTNM-stage	T1-T2	38	40.4		5	27.8		7	38.9		(0.812)
	T3-T4	45	47.9		10	55.6		9	50.0		
	Tx	9	9.6		3	16.7		2	11.1		
	N0	67	71.3		13	72.2		12	66.7		(1.000)
	N1-N2	21	22.4		3	16.7		4	22.2		
	N3	3	3.2		2	11.1		1	5.6		
UICC-stage	Stage I-II	31	33.0		6	33.3		5	27.8		(1.000)
	Stage III-IV	63	67.0		12	66.7		13	72.2		
Histology	SCC	75	79.8		17	94.4		15	83.3		(0.735)
	Other	19	20.2		1	5.6		3	16.7		
Grading	Low Grade (G1-G2)	50	53.2		12	66.7		7	38.9		(0.156)
	High Grade (G3-G4)	41	43.6		6	33.3		11	61.1		
Localization	Oral cavity	24	25.5		7	38.9		4	22.2		(0.308)
	Oro-, nasopharynx	26	27.7		5	27.8		9	50.0		
	Hypopharynx, larynx	6	6.4		2	11.1		1	5.6		
	Lymph node	8	8.5		3	16.7		2	11.1		
	Other	30	31.9		1	5.6		2	11.1		
Resection margins	R0	37	39.4		4	22.2		6	33.3		(0.766)
	R1	32	34.0		10	55.6		5	27.8		
	R2	11	11.7		3	16.7		4	22.2		
	Rx (unsure)	6	6.4		1	5.6		-	-		
	No debulking	8	8.5		1	5.6		3	16.7		
Tissue invasion	L0, V0, Pn0	26	27.7		3	16.7		2	11.1		(0.733)
	L1, V1 or Pn1	20	21.3		5	27.8		7	38.9		
	No neck dissection	48	51.1		10	55.6		9	50,0		(1.000)

Shows the patient and tumor characteristics for the total group, study group receiving the cetuximab-taxane schema and the matched pair control group that potentially influence prognosis. Differences between the study group and the control group were analyzed by *chi-square test and Mann–Whitney U test

Seventeen (94 %) patients were treated with surgery. One of the patients within the treatment group refused tumor reduction. In 4 (22.2 %) patients we achieved a microscopically margin-negative resection (R0). In 10 (55.6 %) patients microscopic tumor mass remained (R_1_). However, in 3 (16.7 %) patients only debulking of the tumor mass (R_2_) was feasible. Fourteen (77.7 %) patients had surgical resection at the primary site and 3 (16.7 %) patients received neck dissection only. In 8 (44.4 %) cases plastic reconstruction was necessary (4 pedicled muscle, 1 microvascular, 3 random pattern flap).

15 patients (83.3 %) were previously radiated by external beam radiation with an average of 68.1 Gy (range 50–105). To exhaust treatment modalities, 4 (22.2 %) patients underwent adjuvant external beam radiation (EBRT) with a unit dose of 2.0 Gy (range 1.8-2.0) in 25.8 fractions (range 25–28), to a total dose of 50.1 Gy. Two patients received further adjuvant chemotherapy.

BT was applied for 7.1 days (range 4–9) with a fraction dose of 2.5 Gy in mean 10.6 fractions (range 6–14), to a mean total dose of 27.0 Gy (range 15–35). On average we used five radiation catheters (range 3–11), using the Paris system geometric rules for implantation.

The radiated clinical target volume (V_100_) was on average 61.9 ml (range 22.3-149.5). We achieved a homogeneous radiation field, with a mean 150-isodose surface (V_150_) of 23.1 ml (range 7.1-55.2). The non-uniformity ratio (DNR), the quotient of V_100_ and V_150_, was 0.35 (range 0.28-0.46).

The control group showed similar features regarding tumor localization, TNM and UICC stage, tissue invasion and resection margins. Furthermore adjuvant treatment (besides the cetuximab-taxane regimen), function preserving tumor debulking and brachytherapy, between the study group and the matched pair control group corresponded (Table 2). Due to the inclusion criteria significant differences in previous recurrence free survival (*p* = 0,003) and age at onset of recurrence disease (*p* = 0,006) were detected. Though not significant, similar distribution in tumor grading was not reached as well.

**Table 2:** Treatment characteristics

		Total group *(n = 94)*			Study group *(n = 18)*			Control group *(n = 18)*			*p*-values*
		n	%	value (range)	n	%	value (range)	n	%	value (range)	
Surgical treatment	Total	88	93.6		17	94.4		15	83.3		(0.279)
	Local resection	78	83.0		14	77.7		14	77.7		(0.397)
	Neck dissection	10	10.6		3	16.7		1	5.6		(0.790)
Brachytherapy	Total dose (Gy)			25.9 (10–35)			27.0 (15–35)			27.1 (20–30)	(0.696)
	Single dose (Gy)			2.5 (2.5-4.5)			2.5 (2.5-3.0)			2.5 (2.5-2.5)	(0.791)
	Fractions			10.2 (4–14)			10.6 (6–14)			10.8 (8–12)	(0.938)
	Radiation days			6.3 (1–11)			7.1 (4–9)			6.6 (3–9)	(0.202)
	V_100_ (ml)			50.0 (3.6-149.5)			61.9 (22.3-149.5)			46.4 (4.0-111.1)	(0.291)
	V_150_ (ml)			19.1 (1.6-65.3)			23.1 (7.1-55.2)			17.0 (2.0-37.3)	(0.347)
	DNR			0.37 (0.32-0.56)			0.35 (0.28-0.46)			0.40 (0.29-0.56)	(0.168)
Adjuvant treatment	EBRT	24	25.5		4	22.2		3	16.7		(0.557)
	Radiation dose (Gy)			48.7 (30–60)			50.1 (50.0-50.4)			35.8 (30–45)	(0.887)
	Chemotherapy (total)	15	16.0		18	100		2	11.1		(0.000)
	Chemotherapy (only platinum derivatives)	5	5.3		2	11.1		-	-		

Gives an overview of the performed treatment modalities. The ‘Cetuximab-Taxane Recurrency Scheme’ combines salvage surgery (SS), Brachytherapy (BT) and adjuvant chemoprotocol in regard to previous external radiation (EBRT), previous chemotherapy (CTx). To exhaust chances an external boost was added whenever reasonable. Differences between the study group and the control group were analyzed by *chi-square test and Mann–Whitney U test

### Disease free survival and overall survival

**Fig. 3:**
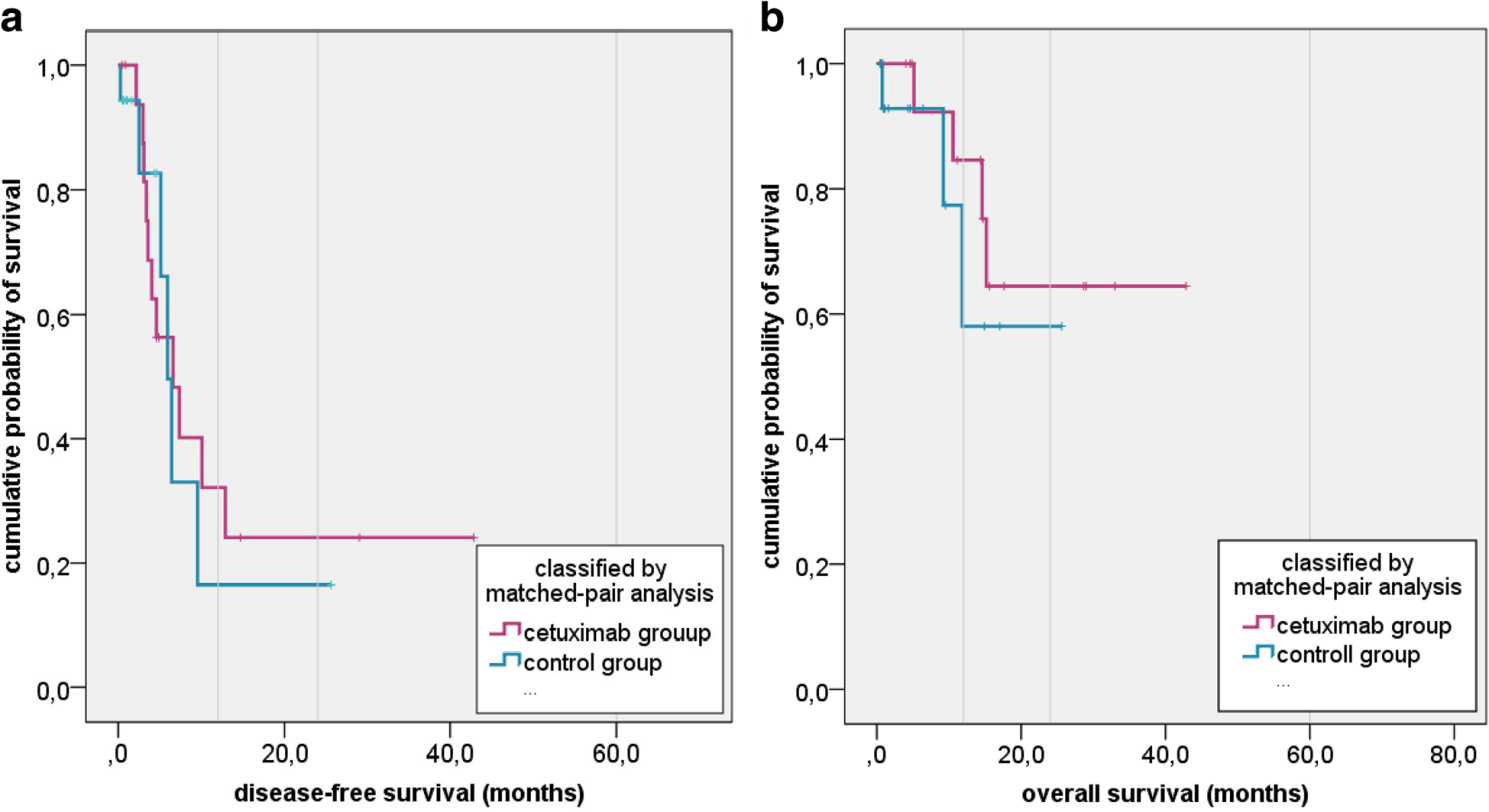
Impact of the cetuximab-taxane recurrency scheme on DFS and OS. **a** displays the disease-free survival (DFS) and **b** the overall survival (OS) of the study group (magenta) and the matched pair control group (blue). DFS enhanced about 4.8 months (*p* = 0.13) and OS about 8.7 months (*p* = 0.023)

Average DFS and OS of the matched pair group, treated by debulking and brachytherapy alone, were 4.0 months (range 0.3-25.6) and 6.2 months (range 0.5-25.6) respectively. The 1- and 2-year OS rates were 68 % and 58 %, respectively, the DFS rates were 17 % each. Whereas, DFS and OS of the study group improved at 8.7 months (range 0.5-42.9) and 14.9 months (range 0.5-42.9) respectively. The 1- and 2-year OS rates were 85 % and 65 %, respectively, the DFS rates were 32 % and 24 %, respectively. Thus, DFS was enhanced by approximately 4.7 months (*p* = 0.13) and OS by approximately 8.7 months (*p* = 0.023) (Fig. 3a-b). Survival benefit reached significance in the paired sample t-test.

In detail, in the study group 11 (61.1 %) patients developed tumor recurrence following the treatment. Seven (38.9 %) patients survived with recurrent disease. Four (22.2 %) patients died of the tumor disease. In the matched pair group 6 (33.3 %) patients developed another relapse after treatment. Four (22.2 %) patients survived with recurrent disease. Two (11.1 %) patients died of the tumor, one patient due to another disease. Although more patients developed recurrence in the study group, the time to re-recurrence was prolonged. The mean follow-up was 13.4 months (range 0.1-72.9).

### Early and late toxicities

Early complications like lymph edema, skin reaction, mucositis or wound healing disturbances were observed in 8 patients of the study group. In most cases they were less severe (grade I-II 33.3 %, grade III 11.1 %). We recorded specific acute toxicities of the cetuximab-taxane protocol, like acneiform rush (11.1 %), hypersensitivity reaction (5.5 %) or myelosuppression e.g. neutropenia (5.6 %), but no postoperative bleeding or peripheral neuropathy.

Late complications like fibrosis, persistent edema or wound healing disturbances occurred in 5 (27.7 %) patients (grade I-II 22.2 %, grade III 5.6 %). Four (22.2 %) patients suffered from persistent dysphagia after treatment.

Compared to the matched pair group, there was no evident increase of therapy induced acute or late toxicities more than grade III. However, mild wound healing disturbances were detected more often in the treatment group (Table 3).

**Table 3:** Treatment related toxicities

	Total group	Study group	Control group	Salvage surgery	Salvage surgery plus BT	BT in palliation	Cetuximab	Paclitaxel
%	(*n* = 94)	(*n* = 18)	(*n* = 18)					
Overall acute and chronic side effects:								
CTC overall	26.6	44.4	33.3	36-39 (III,IV,V)	7-23 (III,IV)	13-35 (II,III)	15-41 (III-IV)	
CTC I	6.4	16.7	5.6					
CTC II	10.6	16.7	16.7					
CTC III	9.6	11.1	11.1					
LENT-SOMA overall	14.9	27.8	11.1		7-23 (III,IV)	7-33 (II,III)		
LENT-SOMA I	3.2	5.6	5.6					
LENT-SOMA II	7.4	16.7	--					
LENT-SOMA III	4.3	5.6	5.6					
Acute and chronic side effects in detail:								
Mucositis	5.4	11.1	5.6		10 (III)	60 (I,II)	8-56 (III,IV)	17-35 (III,IV)
Lymph edema	10.6	16.7 (II,III)	11.1					21
Pain	--	--	--			20	6	
Dysphagia	6.4	22.2	--	53-58 (III)	32-39 (II,III)	4-16	26 (II,III)	15
Bleeding	3.2	--	16.7	1-3 (IV,V)	3-14 (II,III,IV)	3-7 (III)	8 (II,II)	14 (III)
Woundheeling disorder	10.6 (II,III)	16.7 (II,III)	5.6 (II,III)	12-17	15-17 (II,III)	4 (III)	11-23 (II,IV)	
Soft tissue necrosis	--	--	--		7 (III)	7-28 (II,III)		
Osteoradionecrosis	--	--	--		4 (III,IV)	1-17 (II,III)		
Fibrosis	2.1	5.6	--		7-29 (II,III)	2-7 (II,III)		
Skin reaction	3.1	11.1 (III,IV)					9-16 (III,IV)	12
Infusion reaction	1.1	5.5 (III)					2-22 (III,IV)	
Neutropenia	1.1	5.6 (IV)					8 (III,IV)	14-75 (IV)
Renal failure	--	--						7 (III,IV)
Neuropathy		--			4-26 (II,III,IV)	7 (III)	4	7 (III,IV)

Shows the toxicity profile of the study group compared to the total group, the control group and to literature. Other studies treating recurrent HNSCC by salvage surgery [34–36], salvage surgery combined with Brachytherapy (BT) [32, 42–44], single BT in palliation [24, 37, 38, 42, 43, 45, 46] or studies that used Cetuximab [1, 11, 47–52] within their regime were listed using the CTC (common toxicity criteria) and LENT (Late Effects Normal Tissue)-SOMA-classifications. The toxicity levels the percentage values from literature refer to were put in brackets

To assess the toxicity of the treatment it has to be considered, that many patients got tracheostomy, feeding tube and central venous access device preventively to manage possible complications like swelling, bleeding or pain related dysphagia (Additional file 1: Table S1).

### Subgroup analysis

We checked for persisting survival advantage by adjusting the data to ‘age at onset of recurrence disease’ (*p* = 0,006). Comparing the patients of the study group who were younger than 60 years to those of the control group, we still observed lower mortality rates and improved OS in the study group. By contrast, comparing the patients older than 60 years, they did not improve in OS, although still showing lower mortality rates. Adjusting to ‘previous recurrence free survival’ (*p* = 0,003) was not possible as all patients of the control group solely had late recurrences.

**Fig. 4:**
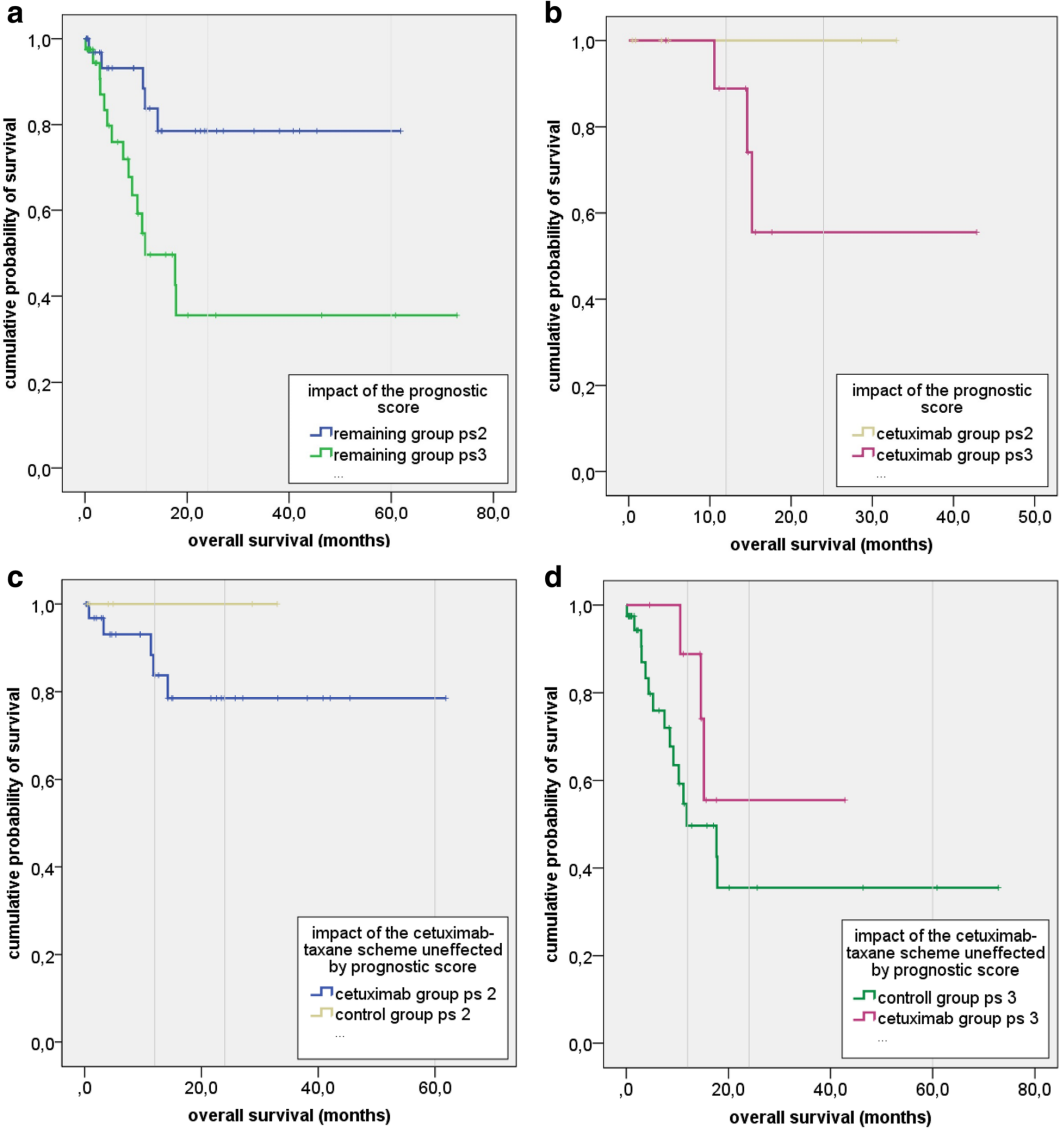
Subgroup analysis. **a** and **b** demonstrate the impact of Chua’s prognostic score on the overall survival of patients with intermediate and bad prognosis in the study group (*p* = 0.3) and the remaining group (*p* = 0.003). **c** and **d** illustrate the impact of the cetuximab-taxane scheme unaffected by the prognostic score. It shows improved survival, but significance could not be demonstrated, neither in patients with intermediate (*p* = 0.4) nor with bad prognosis (*p* = 0.08)

We also examined the impact of further variables on the observed survival benefit. Chua et al. implemented a scoring system for recurrent nasopharyngeal cancer, that predict re-recurrence by age at diagnosis, period between radiation and recurrence, T-stage or tumor volume and number of previous recurrences [9]. We stratified the study group and the remaining total group into intermediate (study group_ps2_, remaining group_ps2_) and bad prognosis (study group_ps3_, remaining group_ps3_). To prove the impact of the cetuximab-taxane scheme, unaffected by prognostic score we compared these groups at the same prognostic level. However still showing improved survival, significance could not be demonstrated, neither in patients with intermediate (*p* = 0.4), nor with bad prognosis (*p* = 0.08) (Fig. 4).

## Discussion

### Treatment regimen

The treatment of recurrent HNSCC is limited by radiation tissue dose, chemotherapy tissue damage, surgical limits and the patients’ performance status. Whenever feasible, salvage surgery is the method of choice with curative intent. Postoperative re-irradiation is expected to increase LC in patients at high risk for local recurrence. However, advantages in OS could not be reported due to accelerated toxicities [20, 21].

A multimodal therapy regimen, containing several tools in anti-tumor treatment, could help to reduce specific toxicity of one treatment but could increase overall destabilization of cancer cells. Recurrent tumor disease often shows higher radiation resistance and higher expression of the epidermal growth factor receptor (EGFR) [22]. Adding the monoclonal antibody cetuximab to the second-line regimen is one option to reduce radio- or chemotherapy resistance.

In well selected patients re-irradiation, administered either with or without concurrent systemic therapy, is feasible [20]. Especially targeted radiation like intensity-modulated radiation therapy (IMRT), predicts better LC and OS compared to conventional techniques [23]. Re-irradiation with HDR-BT shows similar encouraging results, with an acceptable late complication rate of 16 % [24]. BT is known to be a safe alternative of re-irradiation compared to EBRT. Due to 3D conformal and individual optimized treatment planning, treatment related side effects can be kept low. The steep dose-falloff in adjacent tissues spares normal tissue and reduces radiation toxicity [24]. So BT became a useful complement in a multimodal tumor therapy.

Also stereotactic radiation therapy (SRT) has shown to be a reasonable treatment option in recurrent HNSCC and was combined with Cetuximab in palliation, yet [25, 26]. But BT showed better local control and lower toxicity rates than SRT [27]. SRT gives a homogeneous dose distribution, while BT has an inhomogeneous dose distribution. This means that the maximal dose of the SRT applied homogeneous in the target volume is equivalent to the calculated minimal dose applied by BT. Next to the applicator BT can reach dose levels up to 400 percent of this minimal dose. In addition the total irradiated volume is smaller in BT. This leads to less complications like soft tissue necrosis, also when combined with concomitant chemotherapy [28, 29].

Patients not suitable for salvage surgery can be selected for BT combined with simultaneous chemotherapy as an effective and safe option for curative therapy as well [30].

The best in-field control rates in advanced tumors were obtained, when BT was combined with radical surgical excision and plastic reconstruction, using vascularized myocutaneous flaps [31, 32]. Tumor debulking diminishes hypoxic radiation resistant tissue and reduces the target volume [33]. But only a minority achieves long-term survival, because of the high risk of systemic relapse. Especially younger patients, having a disease free interval after receiving definitive therapy or a small recurrent tumor that can obtain negative surgical margins and patients without recurrent neck disease benefit from this treatment [34].

The cetuximab-taxane recurrency scheme combines these strategies. Thereby we enhanced OS to 14.8 months and RFS to 8.7 months. So there is evidence for an additive effect.

### Early and late toxicities

Acute and late toxicities must be taken into account, to value the eligibility of this new regimen as each part of the treatment can cause adverse effects.

First of all, patients are at risk to suffer from transient or permanent perioperative consequences concerning oral intake, speech or breathing, especially in case of salvage surgery. Goodwin et al. reported a rate of 27 % of all perioperative severe complications and a mortality rate of 5.2 %. Frequently problems are cardiopulmonary (4.5 %), bleeding (2.7 %) and wound healing disorders (infection 4.5 %, necrosis 1.8-8.3 % or fistula 5.5-8.3 %) [35, 36].

Secondly, early complications as skin reactions (up to 60 %), pain (20 %) or dysphagia (8-16 %) and late complications as soft tissue necrosis (15-28 %), osteoradionecrosis (0.6-17 %) or chronic woundhealing disorders (3 %) are associated with BT. Although severe complications are rare, carotid blowout is feared especially in previous irradiated tissues [37, 38].

Adding chemotherapy with cetuximab and paclitaxel adds to toxicity. Paclitaxel can lead to myelosuppression, e.g. neutropenia (14 to 75 %), hypersensitivity reaction (2 %), peripheral neuropathy (7 %), mucositis (3 %) and alopecia. Cetuximab is known to cause acute toxicities, like acneiform rush (9-13 %), transfusion reaction (3 %) and disturbance of the water- and salt metabolism. Nevertheless Cetuximab did not show to raise radiation toxicity and is usually well tolerated [1]. Curran et al. showed that mucositis, xerostomia, dysphagia and weight loss did not increase and performance status did not deteriorate [39].

Despite combining these therapy modalities in our investigation, therapy related acute or late toxicities more than grade III did not increase in the study group, neither in comparison to the matched pair control group, nor to literature (Table 3).

### Limitations of the study

As the current investigation is based on retrospectively collected data, the control group had to be built by matched pair analysis. Due to the small number of patients, the power of this study and the validity of subgroup analysis are limited. This is why we would rather speak of a tendency, although we have seen statistical significance.

Small groups lead to difficulties especially in the matching process. We had to decide for some major prognosis factors. So there were differences in the distribution of some factors e.g. the tumor grading. The difficulty in recruiting patients is based on variability of HNSCC recurrences, performance status and compliance of the patients and availability of departments offering BT for HNSCC. Nevertheless, the investigated collective has a realistic broad spectrum of entities and additional treatments.

### Prognostic factors

Specifically the influence of prognostic factors cannot be completely excluded although we used multivariate analysis and subsequently subgroup analysis to confirm our data.

The variety of different factors and scoring systems makes it difficult to figure out which are the most important factors. Besides clinical and histological factors, the metabolism of the cancer cell will be an important focus in the future. High expression of EGFR, as well as negative human papillomavirus (HPV) 16 status, are associated with decreased OS [1]. In this context the authors observed a 13-fold increased risk developing local recurrence [40, 41]. Potentially, accurate knowledge of tumor biology, will not only improve predictive power, but also assists to individual treatment planning.

## Conclusion

The ‘cetuximab-taxane recurrency scheme’ seems to be a valuable complement to the multimodal treatment, which improves OS with tolerable toxicity. Based on the outcome of this investigation, our institution is currently re-evaluating the internal standard treatment regimen for patients with recurrent HNSCC, failing first-line-therapy. The initial promising clinical data provide a good argument to establish this schedule in other medical centers. This justifies a need for prospective randomized clinical trials of a sufficient power. It should be mentioned, that when planning new investigations it is necessary to create a solid base for comparability of the different patient collectives. Therefore an adjusted and generally accepted scoring system has to be developed, that involves molecular as well as clinical prognostic issues, so one can identify those who will benefit most from escalating the treatment.

## Additional file

Additional file 1: Table S1. Preventive complication management. Table S1 shows the distribution of patients having tracheostomy, central venous access device (CVAD) and feeding tube including feeding status at discharge from hospital. (DOCX 36 kb)

## Abbreviations

BT: Brachytherapy
CTC: Common toxicity criteria
CVAD: Central venous access device
DFS: Disease free survival
DNR: Dose non-uniformity ratio
EBRT: External beam radiation therapy
EGFR: Epidermal growth factor receptor
HDR-BT: High-dose-rate brachytherapy
HNSCC: Head and neck squamous cell carcinoma
HPV: Human papillomavirus
IMRT: Intensity-modulated radiation therapy
LC: Locoregional control
LENT-SOMA: Late effects in normal tissues subjective, objective, management and analytic scales
NCCN: National Comprehensive Cancer Network
OS: Overall survival
PF: *Chemotherapy regimen:* cisplatin plus fluorouracil
R_0_: Complete microscopically margin-negative resection
R_1_: Removal of all macroscopic disease, but microscopic margin is positive for tumor
R_2_: Macroscopic residual tumor that was not (complete) resected
RR: Response rate
SRT: Stereotactic radiation therapy
UICC: Union internationale contre le cancer
V_100_: Clinical target volume
V_150_: 150-isodose surface
