# Supplementary material for: Second-line treatment of recurrent HNSCC: tumor debulking in combination with high-dose-rate brachytherapy and a simultaneous cetuximab-paclitaxel protocol
Source: Radiat Oncol. 2016 Jan 20;11:6. doi: 10.1186/s13014-016-0583-0 (PMC4719334; doi:10.1186/s13014-016-0583-0)
Supplement: Additional file 1: Table S1. — Preventive complication management. Table S1 shows the distribution of patients having tracheostomy, central venous access device (CVAD) and feeding tube including feeding status at discharge from hospital. (DOCX 36 kb) [file 13014_2016_583_MOESM1_ESM.docx]

Supplemental Table 1*

|  |  | **Total group**  (n=94) | | **Study group**  (n=18) | | **Control group**  (n=18) | |
| --- | --- | --- | --- | --- | --- | --- | --- |
|  |  | n | % | n | % | n | % |
| **tracheostomy** | yes | 25 | 26.6 | 8 | 44.4 | 7 | 38.9 |
|  | no | 39 | 41.5 | 7 | 38.9 | 3 | 16.7 |
|  | closed | 13 | 13.8 | 2 | 11.1 | 5 | 27.8 |
|  | unknown | 17 | 18.1 | 1 | 5.6 | 3 | 16.7 |
| **CVAD** | yes | 18 | 19.1 | 7 | 38.9 | 2 | 11.1 |
|  | no | 49 | 52.1 | 8 | 44.4 | 9 | 50.0 |
|  | explanted | 5 | 5.3 | 1 | 5.6 | 2 | 11.1 |
|  | unknown | 22 | 23.4 | 2 | 11.1 | 5 | 27.8 |
| **feeding tube** | yes | 32 | 34.0 | 12 | 66.7 | 7 | 38.9 |
|  | no | 41 | 43.6 | 5 | 27.8 | 6 | 33.3 |
|  | explanted | 3 | 3.2 | -- | -- | 1 | 5.6 |
|  | unknown | 18 | 19.1 | 1 | 5.6 | 4 | 22.2 |
| **feeding status** | oral | 51 | 54.3 | 7 | 38.9 | 9 | 50.0 |
|  | feeding tube | 25 | 26.6 | 10 | 55.6 | 5 | 27.8 |
|  | unknown | 18 | 19.1 | 1 | 5.6 | 4 | 22.2 |
